# Factors Impacting Family Caregivers’ Adoption of Digital and AI-Enabled Technologies for Care-Related Tasks

**DOI:** 10.1093/geroni/igaf122.1369

**Published:** 2025-12-31

**Authors:** Niels Wu, Adam Felts, Alexa Balmuth, Chaiwoo Lee, Lisa D’Ambrosio, Samantha Brady, Sophia Ashebir, Joseph Coughlin

**Affiliations:** Massachusetts Institute of Technology, Cambridge, Massachusetts, United States; Massachusetts Institute of Technology, Cambridge, Massachusetts, United States; Massachusetts Institute of Technology, Cambridge, Massachusetts, United States; Massachusetts Institute of Technology, Cambridge, Massachusetts, United States; Massachusetts Institute of Technology, Cambridge, Massachusetts, United States; Massachusetts Institute of Technology, Cambridge, Massachusetts, United States; Massachusetts Institute of Technology, Cambridge, Massachusetts, United States; Massachusetts Institute of Technology AgeLab, Cambridge, Massachusetts, United States

## Abstract

Family caregivers are critical to the delivery of care for older adults, but may experience physical, financial, and emotional strains from the caregiving role. Although existing research has established the capacity for digital technologies to ease these strains, adoption remains limited for some technologies that could support care-related tasks, especially for newer, AI-enabled technologies, indicating a need to explore where caregivers encounter barriers related to usability, cost, availability, or value. In this study, an online questionnaire was fielded to members of the MIT AgeLab’s CareHive Panel, an open membership panel of informal caregivers who either currently provide or formerly provided care to an adult family member. Current caregivers (n = 192) were asked about their reasons for avoidance or cessation of use for four AI-enabled technologies: home monitoring and security systems, digital assistants or smart speakers, medication management platforms or smart dispensers, and health/fitness metric trackers. In general, caregivers who reported high levels of physical and financial strain reported more barriers to technology adoption. Those with lower levels of income and education also reported having more difficulties understanding or using technologies for caregiving. Male caregivers were more likely to report finding technologies to be not useful for caregiving. Caring for an individual with certain conditions such as memory or emotional/mental health problems, or developmental/intellectual disorders, was associated with adoption of fewer technologies. Lastly, living apart from one’s care recipient had a mixed effect on caregivers’ behaviors, being associated with higher levels of adoption for some technologies but lower levels for others.

